# User Engagement and Abandonment of mHealth: A Cross-Sectional Survey

**DOI:** 10.3390/healthcare10020221

**Published:** 2022-01-24

**Authors:** Abdulsalam Salihu Mustafa, Nor’ashikin Ali, Jaspaljeet Singh Dhillon, Gamal Alkawsi, Yahia Baashar

**Affiliations:** 1 College of Graduate Studies, Universiti Tenaga Nasional, Kajang 43000, Malaysia; shikin@uniten.edu.my; 2 College of Computing and Informatics, Universiti Tenaga Nasional, Kajang 43000, Malaysia; jaspaljeet@uniten.edu.my; 3 Institute of Sustainable Energy (ISE), Universiti Tenaga Nasional, Kajang 43000, Malaysia; yahia.baashar@uniten.edu.my

**Keywords:** health behaviour, motivation, gamification, game elements, mHealth, abandonment, continued use

## Abstract

Mobile health (mHealth) apps have great potential to improve health outcomes. Given that mHealth apps have become ubiquitous, there is limited focus on their abandonment. Data concerning crucial metrics, including reasons for adoption and discontinued use, are limited. This study aims to gain broad insights into utilization of mHealth and game-like features promoting user engagement. We conducted a cross-sectional survey of 209 mHealth users worldwide. The 17-item survey assessed sociodemographics, as well as the key motivators for mHealth uptake and discontinued use. Our findings show that sports and fitness activity tracking were the most common categories of health apps, with most users engaging with them at least several times a week. Interestingly, the most downloaded mHealth apps among younger adults include MyFitnessPal, Fitbit, Nike Run Club, and Samsung Health. Critical drivers of abandonment of mHealth apps were amotivation, loss of interest, and experimenting with different apps to identify the most suitable tool. Additionally, the financial cost of mHealth apps is crucial, with most participants advocating for free or more affordable apps. The study findings suggest that while many individuals utilize mHealth, several factors drive their abandonment. Moreover, data indicate that mHealth developers need to consider gamification strategies to sustain user commitment, as well as psychological variables, such as intrinsic motivation.

## 1. Introduction

Mobile health (mHealth) apps are widely used in the health sector to support behavioural health outcomes and improve users’ health. mHealth software runs on smartphones and other mobile devices to promote wellness and prevent medical conditions among the general public [1]. These apps can help shift attitudes and behaviours by disseminating, gathering, and analysing health-related data and supporting interventions. Evidently, mHealth interventions are rapidly growing in popularity. Indeed, 325,000 health apps were widely accessible on major app stores in 2017, up by nearly 24% since 2016 [2,3]. Globally, the mHealth apps market was valued at about USD 28,320 billion in 2018 and is predicted to reach USD 10,235 billion by 2023 [2]. Popular mHealth apps for tracking physical activity (PA), step count, food intake, and medication adherence include Fitbit, Medisafe, Nike+, MyFitnessPal, and Strava.

Several studies highlight the benefits of mHealth [4,5,6]. For instance, patients can self-monitor their progress or set exercise or food intake objectives using health apps. Additionally, these apps can be utilized for online consultations, medication adherence, health literacy, and weight management. Although mHealth apps are perceived as beneficial, it is unclear whether they are critical for users to achieve and sustain long-term health behaviour. Prior studies mainly focused on the intention to use mHealth apps, with limited studies on actual usage. As such, intention to use may not be critical in determining actual usage [7]. Hence, there is need to examine factors that determine actual usage of mHealth. In addition, research related to adherence and abandonment of mHealth apps is also limited.

Gamification introduces game-design elements in order to improve a non-game context and make it game-like [8]. To date, gamification has been applied in several domains to make tasks or activities more gratifying, pleasant, and enjoyable. Some popular domains where gamification is applied include online learning [9,10]; healthcare [11,12]; and tourism [13]. The gamification strategy appears to improve intrinsic motivation by fulfilling individuals’ psychological/emotional requirements through the use of game-like components. Intrinsic motivation refers to performance of an activity solely for the sake of enjoyment, excitement, and interest [14]. Various game-design features utilized in gamified systems include badges, points, leader boards, avatars, challenges, and levels [8,15]. Critical components of gamification strategies are similar to existing health behaviour-change techniques (BTC). Within behavioural literature, BCTs are the primary change agents that inspire people to changing their health-related behaviours, such as PA adoption [16]. BCTs may provide suitable strategies to sustaining health behavioural changes.

Similarly, with gamification, several mHealth apps leverage game-design elements to encourage and sustain health-behaviour outcomes. Although past research suggests that gamification can promote health-behavioural outcomes, the results are inconsistent [17,18]. Correspondingly, these studies were short-term and failed to provide evidence of long-term use of or adherence to mHealth.

Despite the promise of mHealth studies on primary motivation for using mHealth apps, evidence of their adoption and discontinued use are limited. Accordingly, we aim to identify the critical success factors (drivers) and motivational affordances behind user uptake, abandonment, and continued usage of mHealth apps. In addressing this limitation, this study expands on existing literature on the acceptance, adherence to, and abandonment of mHealth towards improving users’ PA behaviour.

The rest of the paper is organized as follows: Section 2 describes the research methodology. Section 3 presents the results, while Section 4 discusses the findings. Finally, the conclusion, limitations, and future directions are presented in Section 5.

## 2. Materials and Methods

### 2.1. Survey Items

We conducted an online cross-sectional study over three months in 2021. The questionnaire was adapted to the extant literature [19,20] and further refined to ensure content validity. Necessary wording changes and validation were performed to fit the context of mHealth usage (Table 1). Subsequently, the questionnaire was pre-tested by two experts to avoid issues with wordings, measurement, and ambiguities.

The survey consisted of fifteen items involving thirteen closed and two open questions. The survey items were categorized into the following: (1) sociodemographic characteristics, including gender and age; (2) types of mHealth used and desired features; (3) motivational affordances; (4) reasons for mHealth app adoption; (5) adherence; and (6) discontinued use. The web-based survey was administered to participants via Google Forms, and all items were only available in the English language. The average completion time was six minutes.

### 2.2. Survey Participants

The survey focused on users of the Android Play Store and Apple Store. Previous related literature targeted Android users; however, we targeted users of both platforms to obtain broader perspectives. The exclusion criteria were non-users of mHealth apps. Participants were recruited by email, SurveyCircle online platform, and Facebook. A summary of the study objective and procedure was provided to participants beforehand. We followed our university’s ethical approval and notification processes to protect the respondents’ well-being and to comply with university standards. Participants voluntarily agreed to participate, and no incentives were offered; all questions were compulsory.

### 2.3. Data Collection

The survey was web-based, using a commercially available survey host (Google Forms https://docs.google.com/forms/, accessed on 1 September 2020). Respondents who agreed to participate in the study were required to give informed consent online (click to agree). All of the data for this study were collected over five months from September 2020 to January 2021. We collected data (17-item survey) from the target group of mHealth users anonymously, with no specific personal data saved. After three months, the survey link was deactivated.

### 2.4. Data Analysis

The responses were downloaded into a spreadsheet and reviewed for accuracy and missing values. The data were coded based on themes and analysed accordingly. We assessed non-response bias by comparing early and late responses in two groups, as suggested by [21]. As a result, non-response bias does not appear to be an issue in this study. We categorized the responses into heavy and light users based on extant literature [19,20,22]. Participants who reported using the mHealth apps regularly were defined as “heavy users”. On the other hand, “light users” indicated using mHealth apps occasionally or irregularly. The classification of mHealth users in this study is given in Table 2.

## 3. Results

### 3.1. Sample Characteristics

A total of 209 responses were included in the study. Specifically, according to the findings, 93 males (44.5%) and 116 females (55.5%) participated in the survey. All participants were aged between 18 and 65 years, with a mean age of 28.8. The majority of respondents (75%) were between 18 and 35 years, with the 65-and-older age group having the least number of respondents (0.5%). In addition, participants were current or former users of mHealth. Most respondents (35.9%) spent 3–5 h per day, on average, using a smartphone, followed by 6–10 h per day (26.8%) and 1–2 h per day (16.3%). Of note, only 5.3% of respondents cited using smartphones regularly. 

Given Android’s more considerable market dominance [23,24], it was expected that most respondents use Android (53.2%), as compared to Apple iOS (46.9%). The data show that most users (81.8%) reported using an average of one to three mHealth apps in the past twelve months. In total, respondents downloaded an average of 128 different mHealth apps. App descriptions and categories were coded based on the health behaviour targeted by the app. Furthermore, we identified ten of the most commonly used health apps (Table 3), accounting for more than half (59.7%) of the more likely apps to be downloaded. As indicated, respondents reported using a range of mHealth apps, with MyFitnessPal the most prevalent (14.8%), followed by Fitbit (7.2%), Nike Run Club (7.2%), and Samsung Health (7.2%). Moreover, participants also reported using several other unfamiliar health apps (*n* = 21). Table 3 presents the ten commonly used mHealth apps in terms of user base.

### 3.2. Characterizing Popular mHealth Apps

**Figure 1:**
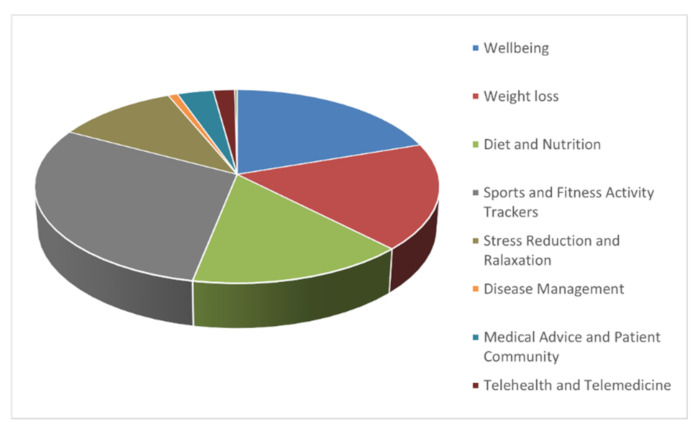
Breakdown of most popular mHealth app categories.

Based on the responses, we considered the most popular mHealth app categories in the mainstream (Apple and Google Play stores), as illustrated in Figure 1. We observed that sports and fitness activity tracking (29.7%), wellbeing (19.8%), and weight loss (17.8%) were the three most popular downloaded app categories. Similarly, regarding features based on a classification of behaviour-change techniques [19,25,26], respondents indicated maintaining or improving physical fitness (34.4%) as the most critical feature. In addition, respondents also revealed maintaining or losing weight (24.9%) and positively changing or improving lifestyle (22%) to be significant features.

As illustrated in Figure 1, respondents identified goal setting (74.6%) as an essential game-like feature. Conversely, activity trackers and step counters are lesser significant features in the mHealth apps. Remarkably, the popularity of the game-like features (points, badges, rewards, and leader boards) shows an increase in consumer acceptance of gamified mHealth apps (30.6%). The increasing recognition of game-like features is shifted from prior literature [19,27], which identified two game features—rewards and sharing—to be less motivating.

### 3.3. mHealth App Adoption

**Figure 2:**
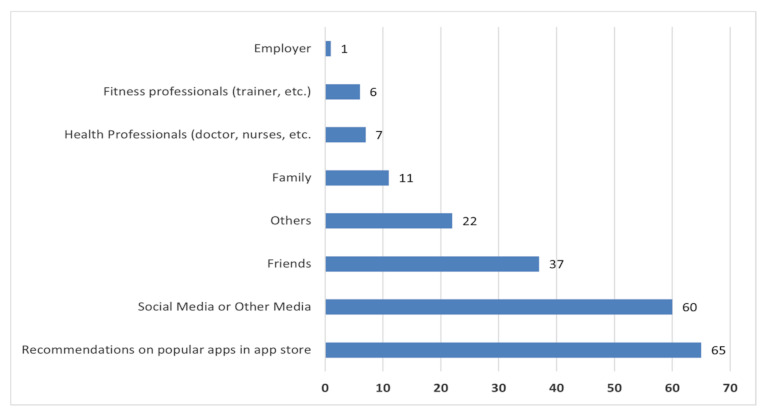
Positive drivers for engaging with mHealth apps.

In the context of mHealth adoption, we explored positive drivers of mHealth app download. We discover that respondents most often discover health apps based on popular recommendations in the app store (Figure 2). However, only a few respondents indicated learning about health apps from their employers or medical professionals. Surprisingly, it is expected that health experts significantly influence recommendations of health apps. Generally, individuals interested in using health apps are expected to seek the advice of medical experts concerning which particular health app to adopt. Such recommendations from medical professionals are usually based on knowledge and experience [28]. However, consistent with previous findings [19], health experts have limited influence on mHealth app recommendations. Moreover, respondents’ positive drivers for engaging with fitness apps include sustaining or improving physical fitness levels, maintaining or losing weight, and improving quality of life.

### 3.4. mHealth App Use

**Figure 3:**
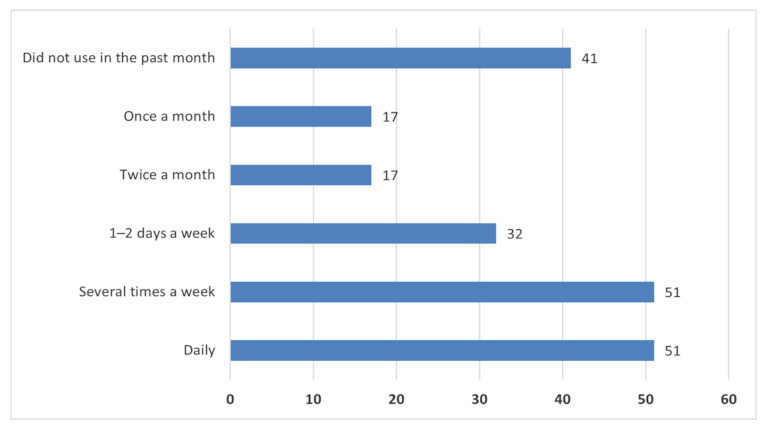
Health app usage frequency.

It is crucial to establish critical factors that promote long-term engagement with mHealth apps. In this context, participants’ primary reasons for engagement were maintaining or improving physical fitness levels (34.4%), losing/managing weight (24.9%), and improving quality of life (22%). Focusing on motivational affordances, we found that respondents are mainly encouraged to use mHealth apps through social media (16.3%) and friends (15.3%). In any case, it should be noted that several users explicitly acknowledged not receiving any encouragement (49.3%). Regarding the frequency of mHealth use, 48.8% of users engage with health apps daily or several times a week (Figure 3). A considerable number of users engaged with an mHealth apps in the past month (19.6%).

### 3.5. mHealth App Abandonment

**Figure 4:**
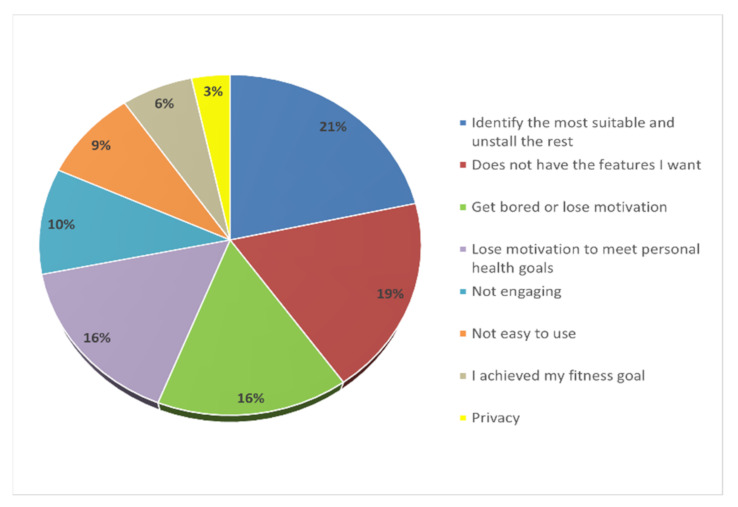
Main reasons for app abandonment by users.

Despite the effectiveness of health and fitness apps, a significant challenge is lack of sustained use. A recent study shows that user commitment to mHealth is low, with about 53% uninstalled within 30 days of download [29]. Figure 4 shows participants’ main reasons for app abandonment. In line with other studies [30,31], the results show that lack of interest or declining motivation (31.6%) is one of the critical factors for the abandonment of mHealth apps. Furthermore, participants highlighted frequently downloading apps before selecting the most acceptable and uninstalling the rest (21.5%). Moreover, lack of desired features in the app (18.7%), the app not being fun (10%), and not being easy to use (8.6%) were also identified as reasons for discontinued use of mHealth apps.

Our results are broadly in line with the literature, which found that the novelty effect significantly influences user abandonment of health-intervention apps [9,15,32]. While users may be drawn to a new app out of curiosity at first, they may lose interest in the app once the novelty effect wears off. As a result, user motivation to use mHealth may decline over time. Therefore, features that sustain healthy behavioural outcomes ought to be identified and implemented in mHealth apps.

Participants were asked what features they would want to see in their existing mHealth apps. From the responses, inductive thematic analysis was adopted to analyse the data, and 15 themes were extracted based on behaviour-change techniques (BCT) specified by the taxonomy of [26]. To assess the implementation of gamification techniques (GT), we used the taxonomy proposed by Hoffmann and colleagues [33]. Table 4 illustrates the 15 themes identified based on BCT and GT. Of note, we observed some overlap between BCT and GT.

The cost of apps was cited as a significant factor among respondents. Interestingly, in line with prior related literature [34,35], most participants advocated that mHealth should be accessible (free) or more affordable. Additionally, limiting of advertisements, more food or dieting options, and the need for more personalized and goal-oriented features were also considered desired features for inclusion in mHealth apps. In gamification context, some identified themes were linked to various motivational affordances (game elements) in mHealth apps. The motivational affordances identified in this study include leader boards, challenges, levels, connection (relatedness), achievement, feedback, prompting, and tracking. An important factor was that most respondents who use non-gamified mHealth apps stated that motivation significantly affects their adherence. Hence, introducing game-like features can increase the prospect of adoption and continued use of fitness apps.

### 3.6. Fitness App Motivation

Motivation is considered critical to the success of mHealth apps. Prior research on health and fitness apps demonstrated that lack of motivation (amotivation) was associated with low adoption of mHealth apps [20]. Accordingly, we examined the role of motivation in adoption of and adherence to mHealth. However, similar to [19], many users were motivated to remain physically active. More importantly, more than half (55%) of the participants perceive that fitness apps motivated them to exercise more, while only 15.3% hold opposite views. In a similar vein, 41.1% concurred that mHealth apps motivated them to eat healthier, while 22.9% disagreed. Furthermore, 73.2% supported that health apps help track their goals, while 8.1% differed.

### 3.7. Heavy vs. Light Fitness-App Users

As previously mentioned, heavy users indicated using fitness apps daily, while participants, who did not use fitness apps within a month, were categorised as light users. This is illustrated in Table 5, along with the participants’ demographic information. Regarding mHealth adoption, heavy users reported using a range of mHealth apps, with MyFitnessPal, Fitbit, Samsung Health, and Strava being the most common. Equally, light users also reported using similar apps. Interestingly, some light users were unable to recall the fitness apps that they installed.

As in Table 5, heavy and light users downloaded the same category of fitness apps: sports and fitness, wellbeing, diet, and weight loss. In the context of motivating features, both user groups reported personalization, food tracker, game-like features, and feedback as the most popular. Besides, light users also reported notifications as a significant feature. Notably, the two user groups considered game-like features (points, badges, rewards, or leader boards) motivating.

Regarding user motivation, most heavy users agreed that mHealth apps inspire them to exercise more (68.6%), eat healthier (52.9%), and keep track of their goals (84.3%). In contrast, 39% of light users disputed that fitness apps encourages them to eat healthier, while 41% were unsure. Also, nearly half (48.8%) of light users concurred that fitness apps encouraged them to keep track of their goals. It is worth noting that over half (53.7%) of light users were unsure whether fitness apps motivated them to exercise more.

Light users identified four reasons explaining why they abandoned mHealth apps. Other participants expressed concern regarding the apps not being enjoyable, absence of desired features, boredom, lack of motivation, or downloading several apps and uninstalling the unsuitable ones.

In the context of features they would like to include in their existing apps, participants reported that apps should be easier to use, more personalized, accessible (free), have more daily notifications, and be more engaging.

Specifically, participants highlighted five prominent examples, summarized below:

- “More simplified, sometimes there’s too many things going on and too many things to track.”
- “Add personalized experiences and feedback”
- “I wish that more of them are free. Or at least less costly. As a student, I don’t really have the money to buy a subscription”
- “Add notifications and measure progress”
- “The thing that lacks in the existing apps is the motivation, it would be perfect to have something that will encourage to continue doing exercise and following the milestones without a break. For now, it is like I use an app for 1, 2, 3 days and then I lose my motivation to do exercise using app.”

## 4. Discussion

In our study, most app users fall into two groups: 18–25 and 26–35 (75%, female: 97, male: 60). Similarly, a previous report showed that the most likely mHealth users in the US are in the 18–34 age group [36]. Notably, as with similar studies [37], older adults have a lower uptake of health apps than younger individuals. Despite low adoption of mHealth apps, recent studies focused on tailored mHealth apps for older adults [38,39]. The increase in the development of novel mHealth targeting the elderly provides an opportunity to improve their PA and fitness levels.

Another critical point is that while subscription cost might affect adoption of mHealth apps, there appears to be a correlation between mHealth pricing and quality. Indeed, more expensive apps tend to be of higher quality in terms of usability and improved features [40]. In other words, free apps may have limited functionality. As a result, this suggests a trade-off between subscription cost and quality of app features.

According to the findings, the two user groups (heavy and light) indicated downloading and using the same category of apps. Additionally, the data suggest that both groups consider similar app features (personalization, food tracker, game-like features, feedback) as critical drivers for uptake and continued use of mHealth. This similarity implies that some fitness-app features may only appeal to highly motivated individuals who are committed and may appeal less to those with lower motivation levels. As such, app developers need to consider personalizing fitness apps to target users with different motivation levels. Additionally, it appears that game elements (leaderboard, badges, points, and challenges) act as motivational affordances, leading to improved app engagement.

More importantly, our data underscores the importance of the novelty effect as a critical factor influencing the lack of sustained use after adoption [32,41]. In a previous study [42], for instance, respondents reported boredom and loss of motivation as reasons for abandoning these apps. These stated factors could be due to lack of interest or enjoyment when using the app after an extended period. As a result, engaging with the apps is no longer fun, and users may not be intrinsically motivated. Hence, the effectiveness of mHealth may not be feasible if users become unmotivated. Further study, for example, utilizing longitudinal designs with a large sample size, is required to effectively examine the correlation between users’ intrinsic motivation and mHealth adherence. This will also ensure increased reliability.

### Implications

Among the most compelling reasons for app abandonment by users is the absence of features that users require. Another primary reason is lack of motivation to sustaining using mHealth apps and maintaining behavioural changes. It becomes critical to involve user input in the design of mHealth apps in order to meet user expectations. The following suggestions are directed to developers and mHealth service providers.

For developers, our findings also have important implications. Indeed, one of the most crucial aspects of app developers’ service offerings is that the app positively impacts health and well-being. Hence, tailoring mHealth apps to the needs of specific user groups seems promising for increasing engagement and preventing a decline in motivation. Nevertheless, developers need to introduce positive strategies and behaviour-change techniques (BTCs) to motivate users to engage with their apps for more significant health benefits.

mHealth service providers should use the findings of this study to include consumer requirements in the app-development process. This would eventually increase consumer engagement levels. The empirically significant factors identified in this study would become the user requirements for mHealth apps. Therefore, mHealth service providers should personalize fitness apps to target users with different types of motivation.

## 5. Conclusions

Gamification of health apps is a promising approach to counteract the often-decreasing long-term motivation of health-app users. Given the rapid adoption of gamification by practitioners and researchers in the healthcare domain, to date, there is little knowledge of the efficacy of mHealth apps in the long term. Although prior studies highlighted the significant impact of motivation on the sustained use of fitness apps [20], it is unclear what drives adoption of, engagement with, and discontinued use of these apps. In response, this study seeks to further explore these gaps. The findings indicated that motivation acts as a positive driver of fitness-app adherence. That is, highly motivated individuals are more likely to continue using health apps and sustain their usage. Reflecting on this point, designers may consider effective strategies to sustain behaviour change in individuals with little or no motivation post-adoption of mHealth apps. We noted, however, that one of these strategies is gamification, essential for promoting continued use. Concerning this, some respondents indicated that boredom affects their long-term engagement with mHealth. Remarkably, respondents advocated introducing game-like features in certain apps to increasing motivation to continue engaging in PA. Thus, the need for further investigation of more effective technology-based solutions for promoting and sustaining healthy behaviour becomes critical.

There are a few limitations to this study. Regarding the checkpoints, recall bias is an issue, as study participants considered mHealth usage in the previous six to twelve months only. Another significant limitation is that we failed to determine the theories applied in the apps because it would have been challenging to ask respondents to self-identify these theories. Notwithstanding, prior research suggests the need for integrative theory-based mHealth apps to sustain health-behaviour change [36,43], primarily studies with longitudinal designs and those centred on integrative theoretical models. While this study was open to all nationalities, we did not ask specific demographic questions identifying respondents’ nationality, race, or profession. In prospective studies, additional demographic questions will improve the cross-cultural generalizability of the outcomes, in particular, sociocultural factors influencing adherence to mHealth apps. Additionally, we failed to identify the direct effect of specific game-design elements on adherence and health-behaviour outcomes.

## Figures and Tables

**Table 1:** Survey measurement items (adapted from [19]).

Items	Measurements
Sociodemographic	What is your Gender?
	What is your Age?
Types of mHealth used and desired features	Which Smartphone Operating System (OS) do you use?
	What kind of health and wellness apps have you downloaded over the past 12 months?
	How many health and fitness apps have you used over the last 12 months?
	What are the most important features for you in the health and fitness app?
Adoption	What is the name of the health and fitness app that you use?
	How do you usually know about the health and fitness apps you install on your phone?
Adherence	What is the main reason you use health or fitness apps?
	How often do you use a health and fitness app in a month?
	What is the main source of encouragement that you receive to use health and fitness apps?
	How many hours do you spend each day on your smartphone?
Discontinued use	If you could change anything about the fitness apps you are using, what would it be?
	Which of these better explains why you stop using the health and fitness apps?
Motivational affordances	Using a health and fitness apps motivates me to exercise more.
	Using a health and fitness apps motivates me to eat healthier.
	Using a health and fitness apps helps me to keep track of my goals.

**Table 2:** Classification of mHealth users.

Classification of User	Usage Frequency
Heavy user	Daily
	Several times a day
	1–2 days a week
Light user	Twice a month
	Once a month
	Did not use in the past month

**Table 3:** Ten commonly used fitness apps.

App	Description	No. of Users	%
MyFitnessPal	Weight loss, calorie counter, and dieting app	31	14.8
Fitbit Health & Fitness	All-day activity, workouts, and sleep-tracking app	15	7.2
Nike Run Club & Training	Running app with GPS-guided run and challenges	15	7.2
Samsung Health	Supports healthier lifestyle, tacks sleep, dieting, and exercise	15	7.2
Strava	App tracking cycling and running with GPS and social networking	12	5.7
Leap Fitness	Full-body workout app	9	4.3
Life Sum Fitness	Personalized dieting, exercise, and calorie-tracking app	8	3.8
Apple Health	Workout, sleep, steps, and all-day activity-tracking app	7	3.3
Flo Fit	Women’s health and fitness and period-tracker app	7	3.3
Calm App	Meditation, sleep, and relaxation app	6	2.9

**Table 4:** Identified themes based on BCT and GT Taxonomies.

Themes Identified	Taxonomy
Cost (More affordable or free)	-
Less in-app advertising	-
Additional food and dieting options	BCT
More personalized (customization)	BCT, GT
Game-like features (combination of game elements)	GT
Use of leader board	GT
Increasing challenges	GT
Level of difficulty (levels)	GT
Social relatedness	GT
Improving motivation	BCT, GT
Syncing with other devices	GT
Improving user experience	BCT, GT
Goal-oriented (achievement)	BCT, GT
Quality of feedback (feedback)	BCT, GT

**Table 5:** Comparison between heavy and light users.

Characteristics	Heavy Users *n* = 51	Light Users *n* = 41
Male	22 (43%)	21 (51%)
Female	29 (57%)	20 (49%)
18–25 age group	19	12
26–35 age group	20	20
36–46 age group	11	8
Android OS	31	21
Apple iOS	20	20
Popular category of fitness apps downloaded	Sports and fitness Wellbeing Diet Weight loss	Sports and Fitness Wellbeing Diet Weight loss
Popular fitness apps	MyFitnessPal, Fitbit, Samsung Health, and Strava.	MyFitnessPal, Samsung Health, Strava, Calm, and 30-day fitness at home.
Most important app features	Personalization, food tracker, game-like features, feedback	Personalization, game-like features, notifications, feedback
Primary motivation for engaging with fitness apps	To maintain or lose weight. To maintain or improve my level of physical fitness. To positively change my lifestyle or improve my quality of life.	To positively change my lifestyle or improve my quality of life. To maintain or lose weight. To maintain or improve my level of physical fitness.
Reasons for abandoning fitness apps	-	Not enjoyable. Does not have the features that I want. Bored or/and lose motivation. Does not meet their demand.

## Data Availability

Data are available from the corresponding author for researchers who meet the criteria for access the data.
